# Deciphering molecular inputs to CRH neurons from POMC neurons

**DOI:** 10.1016/j.fmre.2025.07.001

**Published:** 2025-07-05

**Authors:** Muhammad Junaid, Yiseul Bae, Han Kyoung Choe, Kunio Kondoh, Eun Jeong Lee, Su Bin Lim

**Affiliations:** a Department of Biochemistry & Molecular Biology, Ajou University School of Medicine, Suwon 16499, South Korea; b Department of Biomedical Science, Graduate School of Ajou University, Suwon 16499, South Korea; c Department of Brain Science, Ajou University School of Medicine, Suwon 16499, South Korea; d BK21 R&E Initiative for Advanced Precision Medicine, Ajou University School of Medicine, Suwon 16499, South Korea; e Department of Brain Sciences, Daegu Gyeongbuk Institute of Science and Technology (DGIST), Daegu 42988, South Korea; f Faculty of Medicine, Tottori University, Tottori 683-8503, Japan

**Keywords:** Single-cell transcriptome, Mouse hypothalamus, Molecular interaction, Neurotransmitters, Synaptic activity, Glutamate receptors

## Abstract

Classical neurophysiological studies have highlighted the adaptive role of stress hormones in energy homeostasis and survival-related behaviors in response to intrinsic biological factors and external stimuli. Subsequently, viral tracing research in mice has uncovered that physically restrained, activated corticotropin-releasing hormone neurons (CRHNs) in the hypothalamic paraventricular nucleus (PVN) have direct synaptic input from proopiomelanocortin (POMC) neurons in the arcuate nucleus (ARC), which are classically known to be associated with satiety and autonomic adaptations in metabolic responses. However, the regulatory mechanism, particularly neurotransmitter-based complex connections, still needs to be thoroughly explained. In this study, we have leveraged an integrative approach to comprehend neuron-to-neuron interactions by analyzing publicly available single-cell RNA sequencing (scRNA-seq) datasets and uncovered a dominance of glutamatergic signaling in POMC—CRHNs interactions, which may modulate the autonomic stress response. To explore the evolutionary conservation of this interaction, we further analyzed the human hypothalamic scRNA-seq dataset, confirming that glutamatergic signaling remains dominant with a wider range of ligands from POMC neurons compared to GABAergic signaling. This was further confirmed by viral tracing experiments, indicating that restraint-activated POMC neurons primarily employ significant glutamatergic gene expression. Our study explains molecular mechanisms underpinning glutamatergic signaling in POMC—CRHNs in stress-responsive hypothalamic networks.

## Graphical abstract

**Figure:**
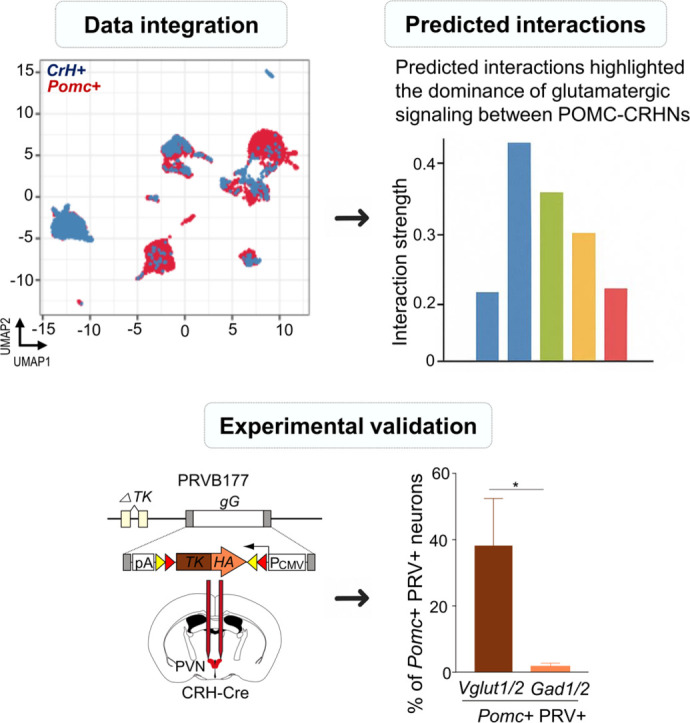

## 1 Introduction

The paraventricular nucleus (PVN) in the hypothalamus contains corticotropin-releasing hormone neurons (CRHNs), which are key regulators of stress hormone secretion [[1], [2], [3], [4]]. Classical neurophysiological and neuroanatomical studies in mice have provided key insights into the functioning of CRHNs and their responses to stress hormones when exposed to a wide range of stimuli [[5], [6], [7]]. These stimuli encompass predator odors, injuries, and various external and internal stressors. Notably, psychological stress induced by physical restraint is also a key regulator of CRHNs [[8], [9], [10]]. More recently, comprehensive insights into anatomical, molecular, and functional inputs to CRHNs have been revealed through viral tracing studies [[10], [11], [12], [13]].

The study utilizing single-cell RNA-seq (scRNA-seq)-assisted viral tracing revealed a specific subset of upstream neurons directly connected to CRHNs [10]. This subset comprised proopiomelanocortin (POMC) neurons located in the hypothalamic arcuate nucleus (ARC), which were previously known for their role in modulating appetite and satiety [[14], [15], [16], [17]]. Interestingly, the study found that physical restraint activated the POMC neurons in the ARC, which are upstream of CRHNs. Chemogenetic experiments were conducted, and the results demonstrated that activating the POMC neurons, similar to physical restraint, led to elevated stress hormone levels. Additionally, chemogenetic silencing of POMC neurons significantly attenuated the stress hormone response to physical restraint. However, the stress hormone responding to the predator odor remained unaffected. These uncovering results imply that POMC neurons, which are known to be associated with satiety, have a significant and specific function in the stress hormone response to a particular sort of psychological stressor—physical restraint—while not influencing the response to another stressor, such as a predator odor [10]. In addition, POMC and CRHNs are also involved in regulating autonomic functions by modulating both endocrine and sympathetic nervous systems, suggesting their role in homeostasis adaptation [[18], [19], [20]].

Which signaling molecules are released by POMC neurons to communicate with CRHNs during specific stress conditions, such as restraint? POMC neurons have been found to release GABA, glutamate, or both neurotransmitters to mediate rapid synaptic signaling, with unique spatial distributions and molecular fingerprints associated with satiety [21]. Additionally, they also secrete neuropeptides such as α-melanocyte-stimulating hormone (α-MSH) that stimulate melanocortin 3/4 receptor (MC3R/MC4R), one of the major neuropeptides involved in satiety regulation, as well as endorphins, endogenous opioids that also regulate the stress response [4,22,23]. However, the specific neurotransmitter-based signaling molecules used by POMC neurons to communicate with CRHNs under stress conditions remain unidentified. To gain a deeper understanding of the molecular signature of POMC neuron-CRHN interactions during stress, it is essential to investigate neuron-to-neuron communication by profiling the neurotransmitter molecules in these interacting cells.

Until now, many computational techniques have been developed to deduce cell-cell interaction networks from coordinated expressions of ligand-receptor interaction pairs [[24], [25], [26], [27], [28]]. Here, we used the R package 'NeuronChat', which combines an inferential model created by integrating prior knowledge on ligand-to-target signaling pathways based on the neurotransmitter, neuropeptides, and synaptic proteins with user-inputted human or mouse data of interacting cells, with a total of 373 interactions [29]. We applied NeuronChat on publicly available scRNA-seq datasets to comprehend the significance of the neurotransmitter-based signaling involved in POMC neuron-CRHN. Notably, the ligand-receptor interactions between POMC neurons and CRHNs indicate the prevalence of glutamatergic signaling, suggesting that POMC neurons predominantly provide excitatory inputs to CRHNs. To validate our findings and assess the cross-species conservation, we extended our study to human hypothalamic data using the NicheNet framework. Similar to mouse data, human POMC neurons also exhibited the dominance of glutamatergic signaling with CRHNs with a broader range of ligand-receptor pairs as compared to GABAergic signaling.

To validate these findings, we employed a viral tracing approach, which confirmed a substantial number of glutamatergic connections within the POMC neurons-CRHNs network. Moreover, our investigation into the upstream POMC neurons of CRHNs activated by restraint stress revealed that these neurons primarily employ glutamatergic markers compared to GABAergic markers. While our study does not directly investigate autonomic regulation, this novel insight provides a comprehensive understanding of the regulatory mechanisms underlying the POMC—CRHNs communication, shedding light on the intricate molecular processes governing this specific stressor-responsive network.

## 2 Materials and methods

### 2.1 Datasets acquisition and integration

To investigate the neuron-neuron interaction between POMC neurons and CRHNs, we utilized publicly available scRNA-seq datasets from both mouse and human hypothalamic studies. We integrated 17 mouse single-cell RNA-seq (scRNA-seq) datasets. This integration includes 10 datasets from earlier work and 7 datasets from Berkhout et al. (2024) [30,31]. These datasets were selected based on the availability of enough gene coverage, standardized metadata, and the inclusion of key hypothalamic regions.

The 10 datasets are from previous work, which had undergone extensive preprocessing using the Seurat package in R (version 4.4.0). Quality control included removing cells with high mitochondrial contamination exceeding 15% and lower than 200 feature counts. Cells were clustered based on neural and non-neural marker genes to identify distinct cell populations. We only retained the cells expressing neuronal genes such as *Snap25, Syp*, and *Syt1* to isolate the neuronal cell population. This filtering resulted in 73,797 high-quality neural cells for further downstream analysis.

We also incorporated 7 PVN-specific datasets from Berkhout et al., due to their high sequencing depth and region-specific enrichment of paraventricular hypothalamic neurons, including CRH-expressing neurons. Unlike other broader hypothalamic datasets, this study included a high-resolution anatomical and molecular profiling of a PVN-specific single-cell transcriptomics atlas. This dataset provides the most comprehensive CRH-expressing PVN neurons, enabling us to model POMC neuron-CRHN interactions with high accuracy. This PVN atlas was already preprocessed, batch-corrected, and specifically enriched with 5545 PVN neurons, ensuing detailed coverage and high sequencing depth.

To integrate the dataset, we employed the Seurat package’s anchor-based workflow. Integration anchors were found by using the "FindIntegrationAnchors()" function with the top 20 primary components. Subsequently, datasets were merged into a single Seurat object "hypo_neurons_17_datasets" using the "IntegrateData()" function. To ensure that the integrated dataset accurately represents the uniform variability and true biological differences, “ScaleData()” was applied to adjust the unwanted variation, such as non-biological noise, sequencing depth difference, or batch effect. Then, to define the clear granularity and prevent the overlapping of similar cells in different clusters, cells were grouped into unique cell populations using the "FindNeighbors()" and "FindClusters()" functions while applying the resolution = 0.25 and 20 principal component dimensions (PCA).

In parallel, we also processed human hypothalamic data using the same pipeline for cross-species analyses [32]. Next, we performed marker-based subsetting of POMC neurons and CRHNs based on gene expression level for both human and mouse datasets separately. Double-positive cells were identified and filtered out to avoid confounding results, allowing for robust analyses. Finally, the subpopulations of CRH+ and POMC+ neurons were merged using the “merge()” function for cell-cell communication analyses.

### 2.2 Neuron–neuron communication

For neuron-neuron interaction analysis of mouse data, we used the NeuronChat R package (version 1.0.0, https://github.com/Wei-BioMath/NeuronChat). NeuronChat utilizes NeuronChatDB, a curated database that includes 373 neural-specific intercellular molecular interaction pairs. Each interaction pair consists of a ligand and a cognate target along with associated genes. By leveraging NeuronChat, we analyzed and visualized the intercellular communication network of neurotransmitter-based signaling between POMC and CRHNs. Using the mouse database, we used the ‘createNeuronChat’ function to create the analytical neuron-chat object. Next, we set the permutation tests with 100 iterations to ensure the robustness of the identified communication pathways to confirm the statistical significance of the interaction. The communication network was then aggregated into matrix form to summarize the interaction strength across POMC—CRHNs, focusing on neurotransmitter-mediated interactions. Interaction strength quantifies the potential signaling activity that is computed from the combined average expression level of ligand-receptors. Then, we utilized the ComplexHeatmap (version 2.20.0) package to visualize the communication pathways with dominant glutamatergic signaling from POMC to CRHNs. For human data, we used the NicheNet framework as it uses an inferential model based on a prior understanding of how ligands influence gene expression in receiver cells, then selects from potential ligands based on receptor expression by the receiver [33]. Gene ontology (GO) enrichment analyses were performed using the R package clusterProfiler (v4.12.6).

### 2.3 Experimental model and subject details

#### 2.3.1 Mice

The Jackson Laboratory provided CRH-IRES-Cre (CRH—Cre; stock no 012704) mice, and The Orient provided C57BL/6J wild-type mice. The Ajou University Institutional Animal Care and Use Committee (2021-0047, 2024-0040) and the DGIST Institutional Animal Care and Use Committee (DGIST-IACUC-25040913-0004) authorized all mouse procedures.

#### 2.3.2 Virus

PRVB177, a pseudorabies virus (PRV) with Cre-dependent expression of hemagglutinin (HA) fused to thymidine kinase (TK), was propagated following the techniques mentioned previously [10,11]. To propagate PRVB177, PK15 cells (American Type Culture Collection) were infected with the virus at 0.1–0.01 multiplicity of infection. Cells exhibited a strong cytopathic impact 2 days after infection. After scraping, the cell material was frozen in liquid nitrogen and thawed in a 37 °C water bath. After 3 cycles of freeze-thawing, cell debris was removed by centrifugation at 1000 *g* for 5 min (repeated twice). The resulting supernatant was used for subsequent studies. The titer of viral stocks was measured on PK15 cells using standard plaque assays [34], with titers expressed in plaque-forming units (pfu).

#### 2.3.3 Stereotaxic injections

According to a previously established methodology, PRVB177 was injected into the PVN of CRH—Cre mice [11]. All injections were performed with 2% isoflurane inhalation anesthesia. In brief, a 1-µL syringe containing 1 µL of PRVB177 (1–1.5 × 106 p.f.u.) was bilaterally injected into the brain at a rate of 100 nL/min, using a Stereotaxic Alignment System (David Kopf Instruments). The needle was carefully positioned into the PVN guided by a stereotaxic atlas (anterior-posterior [AP], −0.4 mm; medial-lateral [ML], ± 0.3 mm; dorsal-ventral [DV], −5.0 mm) [35]. After the treatment, the mice were allowed to recuperate and were housed individually with normal 12-h dark/light cycles, getting food and water as needed.

#### 2.3.4 Restraint stress

Mice were individually restrained in their home cage using a restrainer (a transparent plastic cylinder) [36].

#### 2.3.5 In situ hybridization

In situ hybridization was performed as previously described [12,37], except for a few experiments that required additional steps for triple staining. Fragments of the coding region for *Vglut1, Vglut2, Gad1, Gad2, Pomc*, and *c-Fos* coding and the first intron sequence of *c-Fos* mRNA (for *nFos* labeling) were extracted from mouse brain cDNA or mouse genomic DNA through PCR, then these fragments were then incorporated into the pCR4 TOPO vector (Thermo Fisher Scientific). After that, the ARC brain section of CRH—Cre mice infected for 3 days with PRVB177 or wild-type animals was fresh-frozen in OCT (Sakura) and cut into 20 µm coronal sections using a cryostat. Brain sections were hybridized to Digoxigenin (DIG), dinitrophenol (DNP), and/or fluorescein (FLU)-labeled cRNA probes at 56 °C for 13–16 h.

*Costaining for HA (*PRVB177*), Pomc mRNA, and Vglut1/2 or Gad1/2 mRNAs.* After hybridization, sections were washed twice in 0.2 × SSC at 63 °C for 30 min, incubated with POD-conjugated anti-DIG antibodies (Roche, #11207733910, 1:2000), anti-DNP-KLH antibodies (Molecular Probes, #A6430, 1:200), and biotinylated anti-HA antibodies (BioLegend, #901505, 1:300) at 37 °C for 2 h. Sections were then washed 3 times for 5 min at RT in TNT buffer and then treated using the TSA-plus FLU kit (Perkin Elmer). Sections were then washed 3 times for 5 min at RT in TNT buffer and incubated with 0.5 µg ml−1 DAPI, Alexa555 Streptavidin (Thermo Fisher, #32355, 1:1000), Alexa647 donkey anti-rabbit IgG (Thermo Fisher, #A21447, 1:1000), and at room temperature for 1 h and washed. Sections were mounted with Fluoromount-G *Costaining for POMC, nFos, and Vglut1/2 or Gad1/2 mRNAs.* After hybridization, brain sections were washed twice in 0.2 SSC at 63 °C for 30 min before being incubated for 2 h at 37 °C with POD-conjugated anti-DIG antibodies (Roche, #11207733910, 1:2000). The sections were then washed 3 times for 5 min at room temperature in TNT buffer before being treated with the Perkin Elmer TSA-plus FLU kit. Sections were then washed 3 times for 5 min at room temperature in TNT buffer before being treated with 3% hydrogen peroxide for 30 min to quench POD activity and then rinsed. The sections were then treated with rabbit anti-POMC antibodies (Phoenix Pharmaceuticals, #H-029–30, 1:200) and POD-conjugated anti-FLU antibodies (Roche, #11426346910, 1:300), followed by washing and treatment with the TSA-plus Cy5 kit. After washing, sections were incubated for 1 h at room temperature with 0.5 g ml-1 DAPI and Alexa555 donkey anti-rabbit IgG (Thermo Fisher, #A31572, 1:1000) and then rinsed. Fluoromount-G was used to mount the sections.

#### 2.3.6 Cell counting and statistical analysis

Cell counting was carried out as previously described, utilizing microscopy and a mouse brain atlas for identification [10]. The images were taken with an AxioImager.Z2 microscope and an AxioCam camera. All experiments involved blind counting and analyzing for every fifth section, covering approximately 1.5 mm along the anterior-posterior axis of the ARC. On average, 15 sections per animal were examined. POMC neurons positive for *nFos, Vglut1/2*, or *Gad1/2* were counted manually in each section. The total number of POMC-positive cells and the number of co-labeled cells were summed across all analyzed sections, and the percentage of co-labeled cells was calculated. After checking normal or non-normal distribution, the unpaired *t*-test was used. The data is presented as means ± SEM. Statistical analyses were performed using GraphPad Prism, and unpaired *t*-tests were used. A *P*-value < 0.05 was considered statistically significant.

## 3 Results

### 3.1 Integration and identification of *POMC*+ and *CRH*+ neuronal populations

**Fig. 1:**
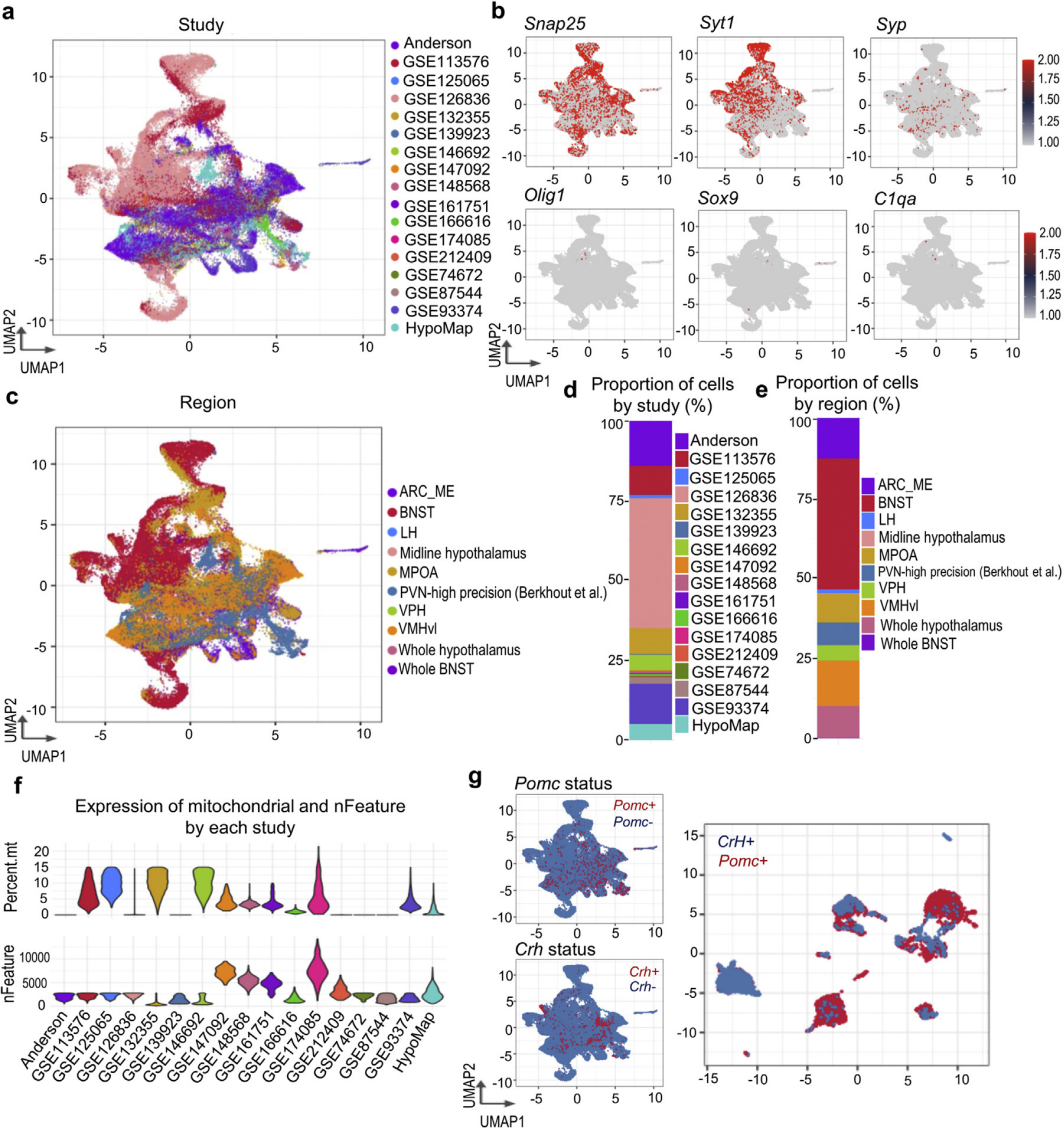
**Identification of POMC neurons and CRHNs from 17 integrated datasets**. (a) UMAP plot visualization of the integrated dataset, with evenly distributed cells colored according to their study of origin. (b) The UMAP plot presents the relative expression of pan-neuronal and non-neuronal marker genes to confirm the neuronal population within the integrated dataset. (c) The UMAP plot with the cells colored by their receptive anatomical region from the hypothalamus. (d, e) The Bar plots provide the proportion of cells by each study and hypothalamic region receptively. (f) The violin plots illustrate the quality control metrics for each study, highlighting that the distribution of mitochondrial gene expression (Percent.mt) is consistently low across studies. Additionally, the number of features (nFeature) affirms the high quality of data. (g) The UMAP plots depict the status of POMC neurons and CRHNs in the integrated dataset, with color-coded UMAP representing the merged POMC+ and CRH+ cells, marked in blue and red, respectively.

We have integrated and reanalyzed datasets from 17 different NCBI GEO studies, covering various hypothalamic regions (Fig. 1a-b). We utilized 10 datasets from previous work, which provided comprehensive coverage of diverse hypothalamic subregions that contain upstream of CRHNs [30]. However, these datasets lacked or had an incomplete representation of neurons from the PVN region, where CRHNs reside. To address this gap, these 10 datasets were integrated with 7 additional high-precision PVN neuron datasets from Berkhout et al., which provided the benchmark for PVN cells with high region specificity and sequencing depth [31] (Supplementary Fig. S1). By highlighting the expression level of the pan-neural markers such as *Snap, Syt1,* and *Syp*, against non-neuronal markers, we confirmed the neural population within the integrated dataset (Fig. 1b). This large-scale integration has provided us with a balanced distribution of neuronal population across each study and hypothalamic region (Fig. 1d, e). The datasets used in this study were implemented with prior rigorous quality control measurements, including filtering out cells with high mitochondrial contamination and normalizing gene expression counts. The quality-controlled measurement shown in Fig. 1f confirms the reliability and consistency of the integrated dataset.

To subset POMC neurons and CRHNs from integrated datasets, we used *Pomc* and *Crh* as biomarkers to identify the POMC+ and CRH+ neurons based on their expression. This marker-based identification allowed us to precisely isolate the specific neuronal population for detailed analyses of their role during specific hypothalamic physiological activity. Then we merged the individual datasets of these cell populations to better understand the intercellular communication between POMC neurons and CRHNs (Fig. 1g).

### 3.2 Cell signaling analysis reveals the predominance of glutamatergic inputs between POMC neurons and CRHNs

To investigate the neurotransmitter-based communication between POMC and CRHNs neuronal populations, we applied the "NeuronChat" package to our merged scRNA-seq dataset. NeuronChat was chosen for this analysis as it is specifically designed to perform quantitative analysis to infer the neuron-neuron communication patterns by considering the specific types of neuronal signaling molecules. It is built on the inferential model to link the gene expression data to neuron-neuron communication probabilities to estimate the coordinated expression abundance of ligand and target [29]. Thus, this approach allows NeuronChat to map the significant communication pattern between POMC neurons and CRHNs through a permutation test.

**Fig. 2:**
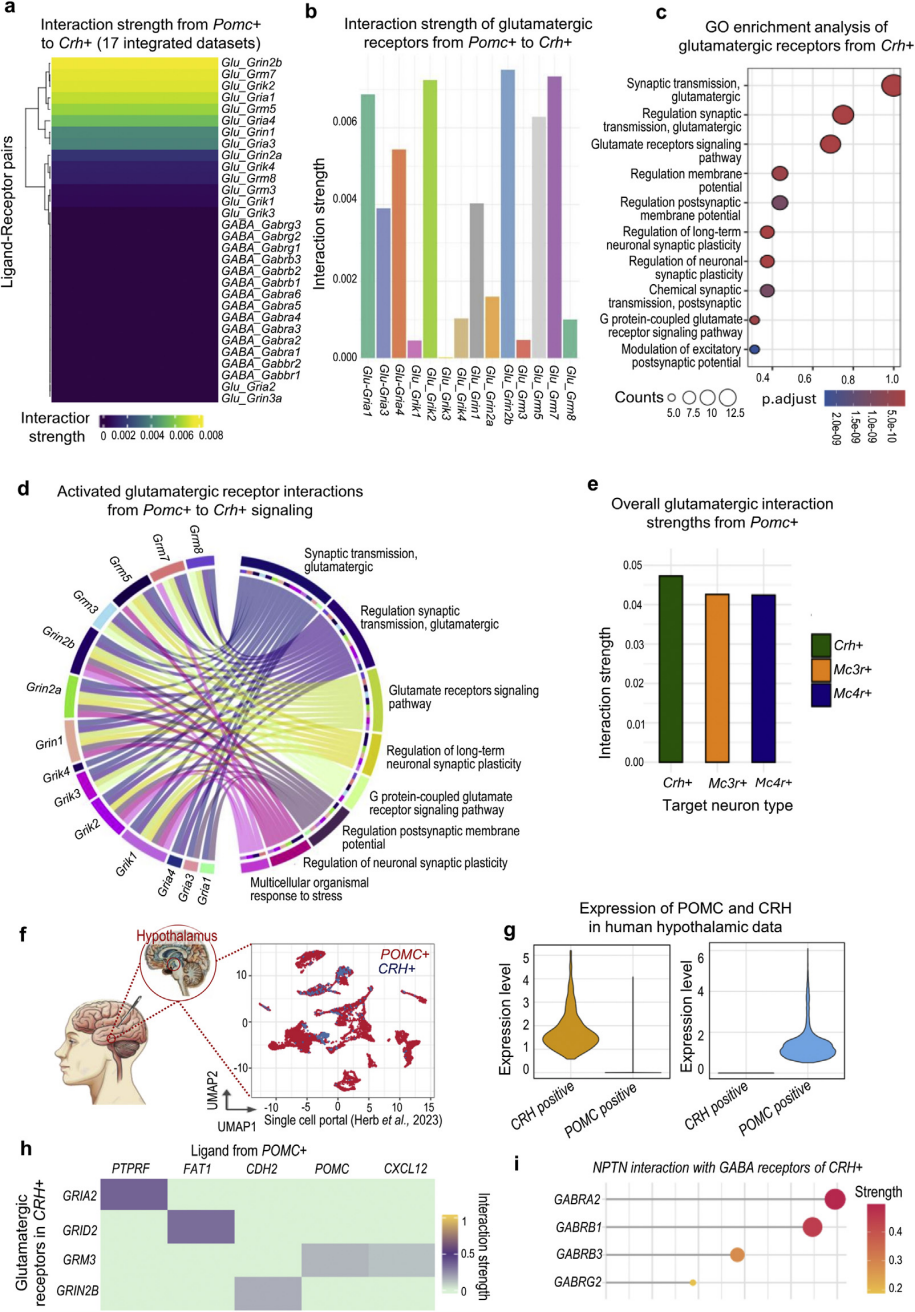
**Neuron-neuron communication analysis revealed the predominance of glutamatergic signaling between POMC neurons and CRHNs**. (a) Heatmap displays the interaction strength of ligand-receptors from POMC neurons to CRHNs in mouse hypothalamic data; the color intensity indicates the strength of the interaction by glutamatergic receptors. (b) The bar plot quantifies the interaction strength of glutaminergic receptors. (c, d) GO results show enriched biological processes of glutamatergic receptors from CRHNs, followed by their interaction with these receptors. (e) The bar plot summarizes the overall glutamatergic interaction strength from POMC neurons to CRHNs or MC3/4R neurons, with each color representing the target neuron types. (f) The UMAP plot shows the merged population of POMC+ and CRH+ neurons from the human hypothalamic dataset. (g) Left panel: violin plot shows the marker expression of CRH+ neurons. The right panel shows the marker expression of POMC+ neurons. (h) Heatmap showing the dominance of glutamatergic signaling with multiple ligands, and (i) the lollipop plot shows GABAergic ligand-receptor interactions with only one ligand.

Our primary focus was to see the communication pattern mediated by glutamate and gamma-aminobutyric acid (GABA), 2 critical neurotransmitters in the hypothalamus. We applied NeuronChat to prioritize which neurotransmitter ligands from POMC neurons, sender cells, are most likely to have impacted the expression of genes in interacting CRHNs, receiver cells. After computing the information flow for each interaction pair of neurotransmitters, our analyses ranked the predominance of glutamatergic signaling by POMC neurons to communicate with CRHNs, suggesting that glutamate/excitatory pathways play a crucial role in intracellular communications (Fig. 2a, b). The bar plot particularly highlights the quantitative interactions involving NMDA (N-methyl-D-aspartate) glutamate receptors such as *Grin2b,* which exhibit stronger signaling strength than other subunits *Grin1* and *Grin2a.* The strong *Grin2b* signaling suggested that POMC neurons may regulate CRHNs by modulating synaptic transmission and plasticity. In addition to NMDA receptors, the presence of other glutamate receptors, including metabotropic receptors *Grm5* and *Grm7*, ionotropic receptors *Gria1, Gria3*, and *Gria4*, and kainate receptor family members *Grik2* and *Grik1,* also underlines the importance of glutamate signaling in these interactions [38,39]. To further understand the functional distinctions of these glutamatergic receptors, we performed gene ontology (GO) enrichment analyses to identify the enriched biological terms (Fig. 2c). The significant enrichment terms are involved in synaptic transmission, regulation, and signaling pathways, followed by complex interaction with these receptor genes and their associated biological functions (Fig. 2d). Moreover, additional receptors from the neuroligin (*Nlgn*) family, which are cell-adhesive proteins, were identified, further highlighting the complexity of POMC neuron-CRHN interactions (Fig. S2a) [40]. Interestingly, our findings show a significant lack of GABAergic signaling, indicating that inhibitory communication between these neuronal populations may be constrained or take place through indirect methods (Fig. S3).

Furthermore, we expanded our analyses to investigate the interactions of POMC neurons with melanocortin receptors MC3/4R, which have been defined as regulators of metabolic homeostasis and stress-induced anxiety behavior [3,41]. Reports also indicate that MC4R signaling activates the hypothalamic-pituitary-adrenal axis (HPA axis) by providing a functional and anatomical connection leading to the release of CRH [3,42,43]. Moreover, POMC neurons from the ARC region release alpha-melanocyte-stimulating hormone (α-MSH), which projects toward MC4R in the PVN region during restraint stress [44]. Several glutamate signaling receptors were notable between POMC neurons and MC3/4R neuronal interactions; specifically, ionotropic receptor subunits (*Gria2* and *Gria1*) are involved in fast glutamate signaling (Supplementary Fig. 2b-d). Notably, POMC neurons have shown glutamatergic signaling with CRHNs and MC3/4R neurons, with the strongest strength with CRHNs, indicating a direct key role in stress-related signaling (Fig. 2e).

To validate our findings about glutamatergic signaling in POMC—CRHN neurons from mouse data, we utilized the publicly available scRNA-seq dataset of human hypothalamus [32]. We subsetted the POMC+ and CRH+ neurons based on their marker expression and performed neuron-neuron communication analyses using the NicheNet framework to infer ligand-receptor interaction (Fig. 2f, g). Notably, the human hypothalamus has also shown the dominance of glutaminergic signaling with a higher number of ligands from POMC neurons targeting CRH neurons based on glutamate receptor expression as compared to GABAergic interaction (Fig. 2h, i).

### 3.3 PVN-neuron-enrichment uncovers predominant glutamate signaling to CRHNs

**Fig. 3:**
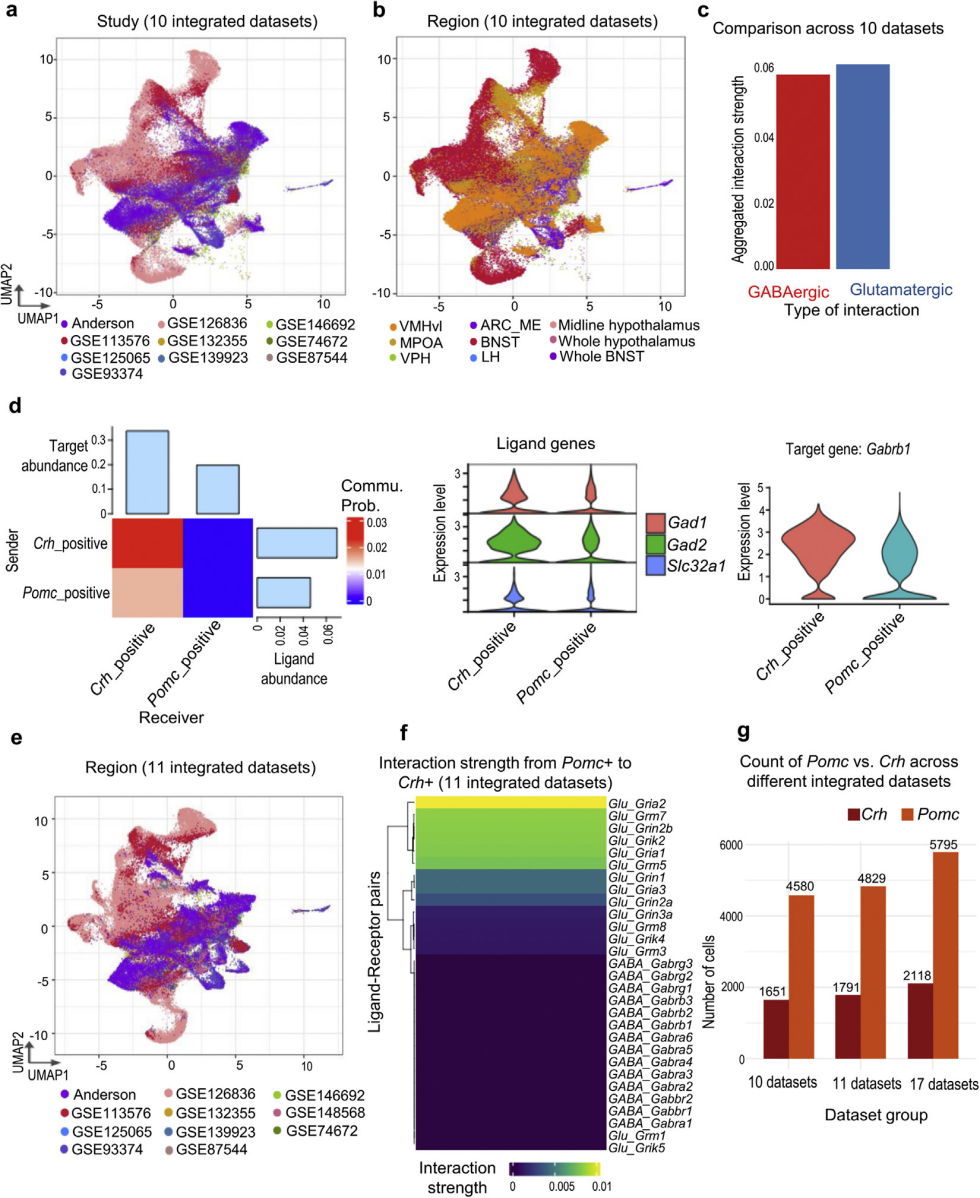
**Increased PVN dataset Integration enhances glutamatergic interaction of POMC neurons with CRHNs**. (a-b) The UMAP plot displays cells from 10 integrated datasets (excluding solely PVN datasets), colored by each study and region. (c) The bar plot compares the aggregate strength of GABAergic and glutamatergic ligand-receptor interactions across 10 integrated datasets, suggesting the significant presence of both types of neurotransmitter signaling. (d) Comparative analyses of GABAergic signaling between POMC neurons and CRHNs show that ligand abundance for GABAergic signaling in POMC neurons is low. At the same time, CRHNs exhibit high expression of the GABA receptor gene *Gabrb1*. (e) The UMAP plot depicts the cells from 11 integrated datasets (adding one PVN dataset to the previous 10), colored by each study. (f) Heatmap showing the dominant glutamatergic signaling from POMC neurons to CRHNs across integrated datasets. (g) The bar plot represents the quantitative number of POMC neurons and CRHNs across 3 integrated dataset groups: 10, 11, and 17 integrated datasets.

Using 10 datasets from previous work, which lacked a comprehensive number of neurons from the PVN region of the hypothalamus, since we focused on the upstream neurons of CRHNs rather than CRHNs of the PVN itself [30], we isolated the POMC neurons and CRHNs for the neuron-neuron interaction (Fig. 3a, b). Our analyses revealed both GABAergic and glutamatergic signaling between POMC neuron-CRHN interactions, in the absence of the PVN region across 10 datasets (Fig. 3c, Fig. S4a). On further exploration of glutamatergic or GABAergic interaction pairs individually, we examined the abundance of ligands and receptors (Fig. 3d). Notably, the data indicates low expression of GABA-related genes in POMC neurons, suggesting reduced GABAergic signaling strength by POMC neurons to CRHNs. In contrast, the target gene for GABA signaling, *Gabrb1*, is highly expressed in CRHNs.

Next, to address the incomplete presentation of PVN neurons, we only integrated the GSE148568 dataset, which offers a detailed and accurate depiction of PVN neurons [45] (Fig. 3e, Fig. S4b). The reanalysis of these 11 integrated datasets revealed more predominant glutamatergic signaling between POMC neuron-CRHN interactions (Fig. 3f). This finding contrasts with 10 datasets, where both GABAergic and glutamatergic interactions were observed. Thus, the increased representation of the PVN region elevated the glutamatergic signaling in the interactions between POMC neurons and CRHNs (Fig. 3g).

### 3.4 Restraint stress activates glutamatergic POMC neurons upstream of CRHNs

**Fig. 4:**
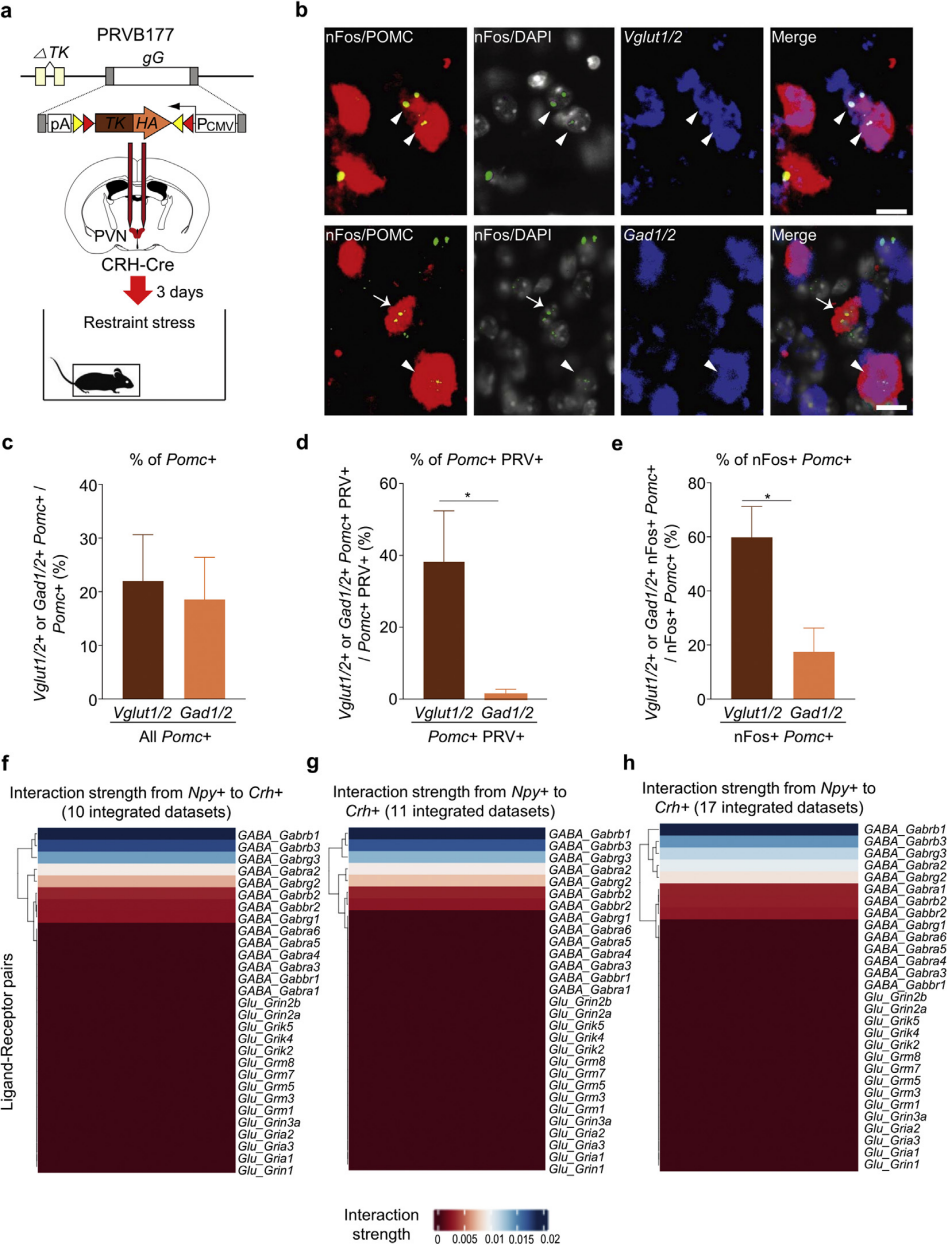
**Experimental analysis reveals the dominance of glutamatergic markers' expression in POMC-CRHNs neuronal communication during stress response**. (a) The schematic illustrates that PRVB177 was injected into the PVN of CRH-Cre mice to infect CRHNs, and the mice were exposed to physical restraint stress on d3pi when the direct upstream neurons of CRHNs were labeled with PRVB177. (b) The ARC sections of physically restrained mice were co-stained for activated neurons (*nFos*) with POMC as well as markers of glutamatergic (*Vglut1/2*) and GABAergic (*Gad1/2*). Nuclear *Fos* detection using riboprobes often reveals 2 distinct punctate signals per nucleus, corresponding to active transcription sites at both alleles of the *Fos* gene. Photographs show ARC sections (one per row) co-stained for POMC (red), *nFos* (green), DAPI (white), and *Vglut1/2* or *Gad1/2* (blue). Arrowheads indicate cells co-labeled for *nFos*, POMC, and either *Vglut1/2* or *Gad1/2*, while the arrow marks a cell positive for *nFos* and POMC but negative for *Gad1/2*. Scale bar, 10 µm. (c) The bar plot shows the quantification of POMC neurons expressing *Vglut1/2* and *Gad1/2* markers across all POMC neurons (n = 6). (d, e) The bar plots show that PRV-labeled and *nFos*-positive POMC neurons predominantly express glutamatergic markers, with error bars indicating S.E.M. *P < 0.05, *t*-test (*n* = 6). (f-h) Heatmap plot showing the high interaction strength of GABAergic signaling from NPY neurons to CRHNs across all 3 integrated datasets.

Previously, it was reported that POMC neurons have a significant impact on how stress hormones react to a particular sort of psychological stressor, restraint [10]. To investigate the activation of glutamatergic POMC neurons upstream of CRHNs in response to restraint stress, we infected the CRHNs of CRH—Cre mice with PRVB177, a Bartha strain of pseudorabies virus (PRVB) with Cre-dependent expression of hemagglutinin (HA) linked to thymidine kinase (TK), which is essential for the replication and spread of PRV [12] (Fig. 4a). The third day after infection (d3pi), this virus identifies directly presynaptic neurons as it moves retrogradely across synapses, allowing it to trace the neural connections [10,12]. Anti-HA antibodies (PRV) and a riboprobe were then used to co-stain sections across the ARC to look for excitatory glutamatergic cell markers (*Vglut1/2*) or a riboprobe for markers of inhibitory GABA neurons (*Gad1/2*), as well as antibodies or riboprobes for POMC (Fig. 4b). Despite comparable proportions of excitatory and inhibitory POMC (both PRV- and PRV+) (Fig. 4c), a majority of PRV-infected POMC neurons, which are directly upstream of CRHNs, are glutamatergic (*Vglut1/2*+) (Fig. 4d). Notably, restraint-activated (*nFos*+, nuclear *Fos* (activated neuronal maker) +) [46,47] POMC neurons are predominantly glutamatergic (*Vglut1/2*+) rather than GABAergic (*Gad1/2*+) (Fig. 4e). Consequently, it can be inferred that during restraint, the upstream POMC neurons of CRHNs primarily facilitate glutamatergic signaling, aligning with findings from a prior chemogenetic study that showcased the role of excitatory inputs from POMC neurons to CRHNs in the augmentation of stress hormone production under restraint conditions [10]. These findings collectively indicate that the excitatory inputs from POMC neurons have the potential to activate CRHNs in response to physical stress, representing a noteworthy stride towards unraveling the molecular intricacies of the POMC neurons and CRHNs neural circuitry.

Additionally, we also analyzed the neurotransmitter-based interaction of neuropeptide Y (NPY) with CRHNs, which are known to antagonize POMC neurons [48]. Interestingly, NPY neurons also exert a regulatory influence on CRHNs, impacting stress response and various endocrine pathways [49]. In all 3 integrated 10, 11, and 17 datasets, we observed the dominance of GABAergic signaling, indicating the consistent inhibitory role of NPY in modulating CRHNs activity that further contrasted with the excitatory role of POMC neurons (Fig. 4f-h).

## 4 Discussion and conclusion

Stress hormones regulated by hypothalamic CRHNs have complicated physiological effects on both the brain and body, and their dysregulation is a major risk factor for neuropsychiatric illnesses such as melancholy, mood disorders, anxiety, and autonomic functions. Emerging evidence indicates that appetite-regulating neurons, particularly POMC neurons in the ARC, also play a pivotal role in modulating stress responses. Notably, POMC neurons are activated by certain psychological stressors, such as physical restraint, and their activation can induce stress hormone release. Conversely, silencing these neurons markedly reduces the stress hormone response to such stressors, highlighting a direct functional connection between metabolic and stress-regulatory circuits [10]. Here, we investigated the neurotransmitter-based functional connectivity of POMC neurons and CRHNs by profiling the scRNA-seq data with viral tracing approaches.

Previous studies have shown that POMC neurons have an excitatory influence on PVN neurons via α-MSH and MC4R signaling [50]. Leptin, an adipocyte hormone, regulates the POMC neuronal activity by suppressing the inhibitory neuropeptide signaling, thereby maintaining the excitatory inputs to PVN neurons [51]. Our findings also align with the excitatory model and further demonstrate that POMC neurons modulate CRHNs activity through glutamatergic transmission, thereby extending the functional role of POMC neurons from metabolic regulation to stress hormone control.

Using NeuronChat and the NicheNet framework, we mapped the ligand-receptor interactions between POMC neurons and the CRHNs. Our research indicated that mostly G protein-coupled receptors (GPCRs) and NMDA receptors for glutamate of CRHNs show the predominance of glutamatergic signaling between POMC neurons and CRHNs, indicating a potential mechanism underlying stress-induced hormone. To extend these findings and explore the evolutionary conservation, we also performed cross-species analyses on publicly available human hypothalamic scRNA-seq data, providing valuable evidence of glutamatergic signaling and contributing to a deeper understanding of hypothalamic neurocircuitry. While our study does not directly include data from individuals with stress-related disorders in the human brain, it confirmed the preservation of glutamatergic signaling between POMC neuron-CRHN interactions.

To validate computational results, we further conducted viral tracing experiments using PRV in CRH—Cre mice, which allowed us to trace the upstream POMC neurons activated by CRHNs during restraint stress. We observed that the percentage of excitatory glutamatergic neurons in the *Pomc*+ PRV+ populations was much higher than that of inhibitory GABAergic neurons following physical restraint. These results support that POMC neurons provide direct excitatory input to CRHNs in response to physical constraint. Our findings align with previous work, where we used chemogenetic activation and silencing to demonstrate the response of POMC neurons to stress hormone during physical restraint but not predator odor [10]. Using the NeuronChat prediction-based model on our integrated scRNA-seq dataset, we also explored neurotransmitter-based signaling pathways between NPY neurons and CRHNs, indicating the significant inhibitory interplay between NPY neurons and CRHNs, in contrast to the excitatory interaction between POMC neurons and CRHNs. These computational results highlight the utility of transcriptomic data in revealing distinct molecular interactions, but also underscore important limitations, such as functional or synaptic connectivity at the protein level. Nonetheless, the significance of our study extends beyond circuit mapping, providing insights into how specific neuronal inputs influence endocrine response under specific stress conditions.

Overall, our integrative approach that combines computational analysis with virus tracing methods offers novel perspectives on the intricate neurotransmitter-based communication network between POMC—CRHNs, which is required for stress hormone responses under a specific type of stressor, restraint. Using similar approaches, it should now be possible to dissect the molecular and anatomical basis of stress responses to various types of stressors. We believe our study may have implications for future studies aiming to integrate molecular and functional data into anatomically defined neural circuit maps relevant to both normal physiology and disease.

## Data and code availability

The normalized datasets of mouse and human, and R codes used for the analyses to produce relevant figures and plots, can be found at Figshare, (https://doi.org/10.6084/m9.figshare.24086037.v4) [52]. Seurat [53] package in R was used for data processing, and the NeuronChat R package (version 1.0.0, https://github.com/Wei-BioMath/NeuronChat) to facilitate neuron-to-neuron signaling analysis.

The source datasets used in this paper are collected from the following database record, for 10 integrated datasets from Junaid *et al*., 2023: Figshare, (https://doi.org/10.6084/m9.figshare.21981251.v1) [30], for 7 PVN high precision datasets from Berkhout *et al*., 2024: Zenodo, (https://doi.org/10.5281/zenodo.8160037).

## CRediT authorship contribution statement

**Muhammad Junaid:** Writing – review & editing, Writing – original draft, Visualization, Validation, Methodology, Formal analysis. **Yiseul Bae:** Validation, Methodology. **Han Kyoung Choe:** Validation, Supervision, Methodology. **Kunio Kondoh:** Validation, Methodology. **Eun Jeong Lee:** Writing – review & editing, Validation, Supervision, Methodology, Funding acquisition, Conceptualization. **Su Bin Lim:** Writing – review & editing, Supervision, Funding acquisition, Conceptualization.

## Declaration of competing interest

The authors declare that they have no conflicts of interest in this work.

## Appendix Supplementary materials

Image, application 1
