# Supplementary material for: Deciphering molecular inputs to CRH neurons from POMC neurons
Source: Fundam Res. 2025 Jul 5;6(3):1975–84. doi: 10.1016/j.fmre.2025.07.001 (PMC13247476; doi:10.1016/j.fmre.2025.07.001)
Supplement: Supplementary file 1 [file mmc1.pdf]

Table of contents for each supplementary figure

**Title**	**Slide no,**
Supplementary Figure. 1	2
Supplementary Figure. 2	3
Supplementary Figure. 3	4
Supplementary Figure. 4	5

a

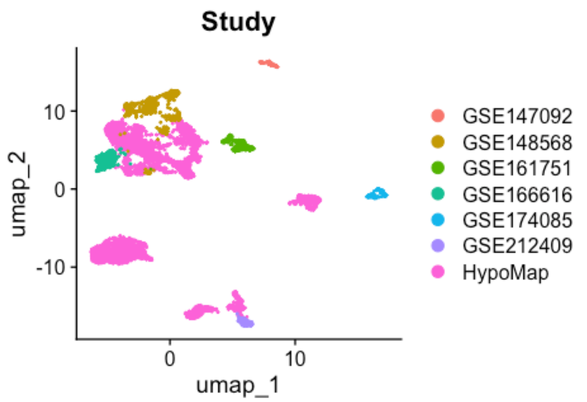

b

Datasets	No of PVN neurons
GSE147092	97
GSE148568	630
GSE161751	247
GSE166616	333
GSE174085	118
GSE212409	142
HypoMap	3978

c d

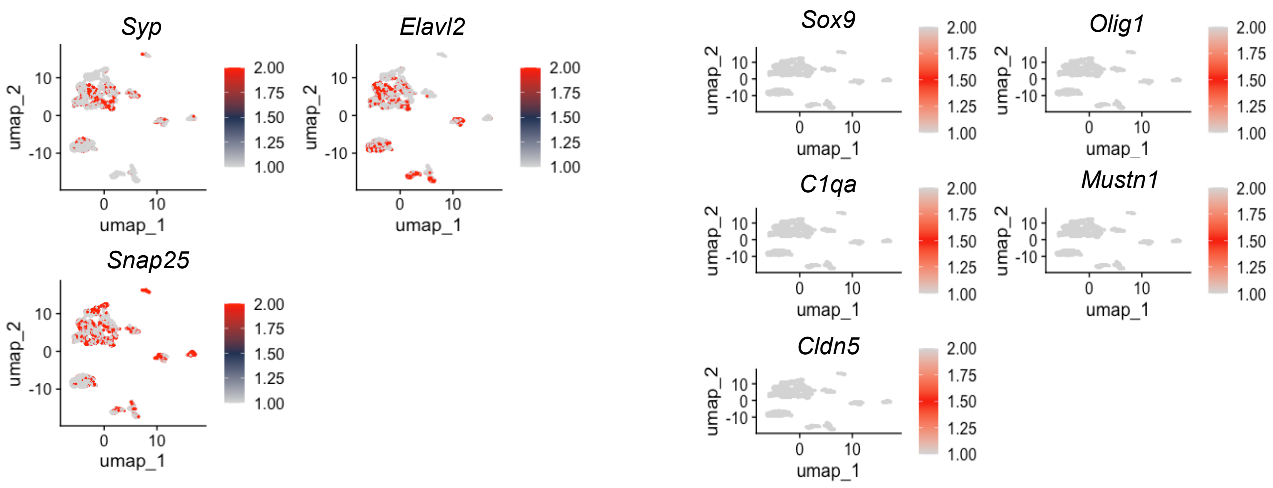

Figure S1. Characteristics of highly precise PVN neurons from Berkhout et al. (a) UMAP plot showing the integrated population of PVN neurons from 7 datasets. (b) The table lists the QC-passed PVN neurons identified in each dataset. (c-d) UMAP plot showing the presence of only neuronal cells by comparing the expression level of pan-neuronal and non-neuronal markers.

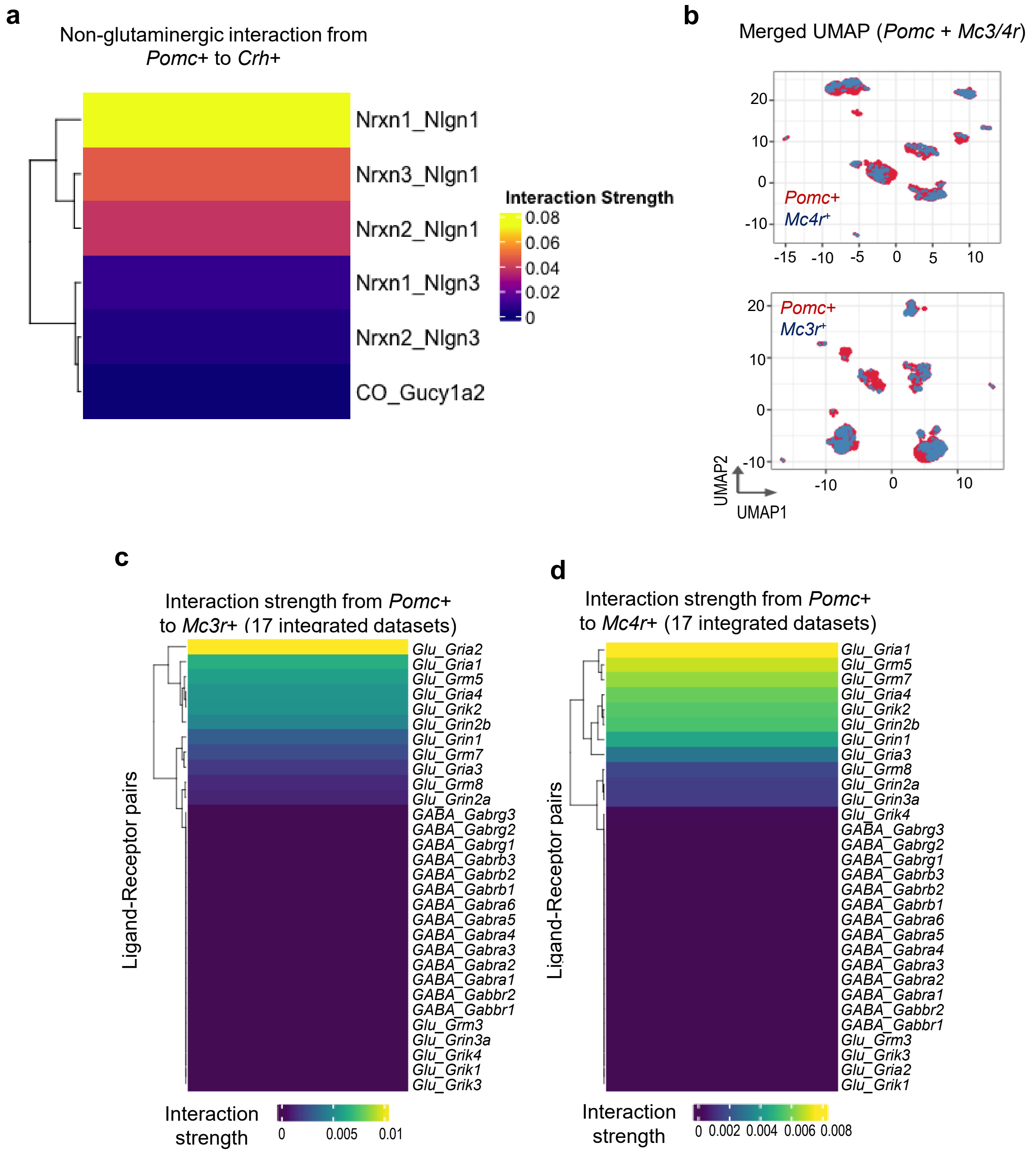

Figure S2. Multiple interactions between mouse hypothalamic POMC-CRHNs and POMC-MC3R/MC4R neuronal communication. (a) The heatmap shows the interaction strength of non-glutaminergic interactions between POMC-CRHNs. (b) Merged UMAP plots visualizing Pomc with Mc3r/4r. (c-d) Heatmaps showing the interaction strength of ligand-receptors from POMC neurons to MC3/4R neurons, respectively.

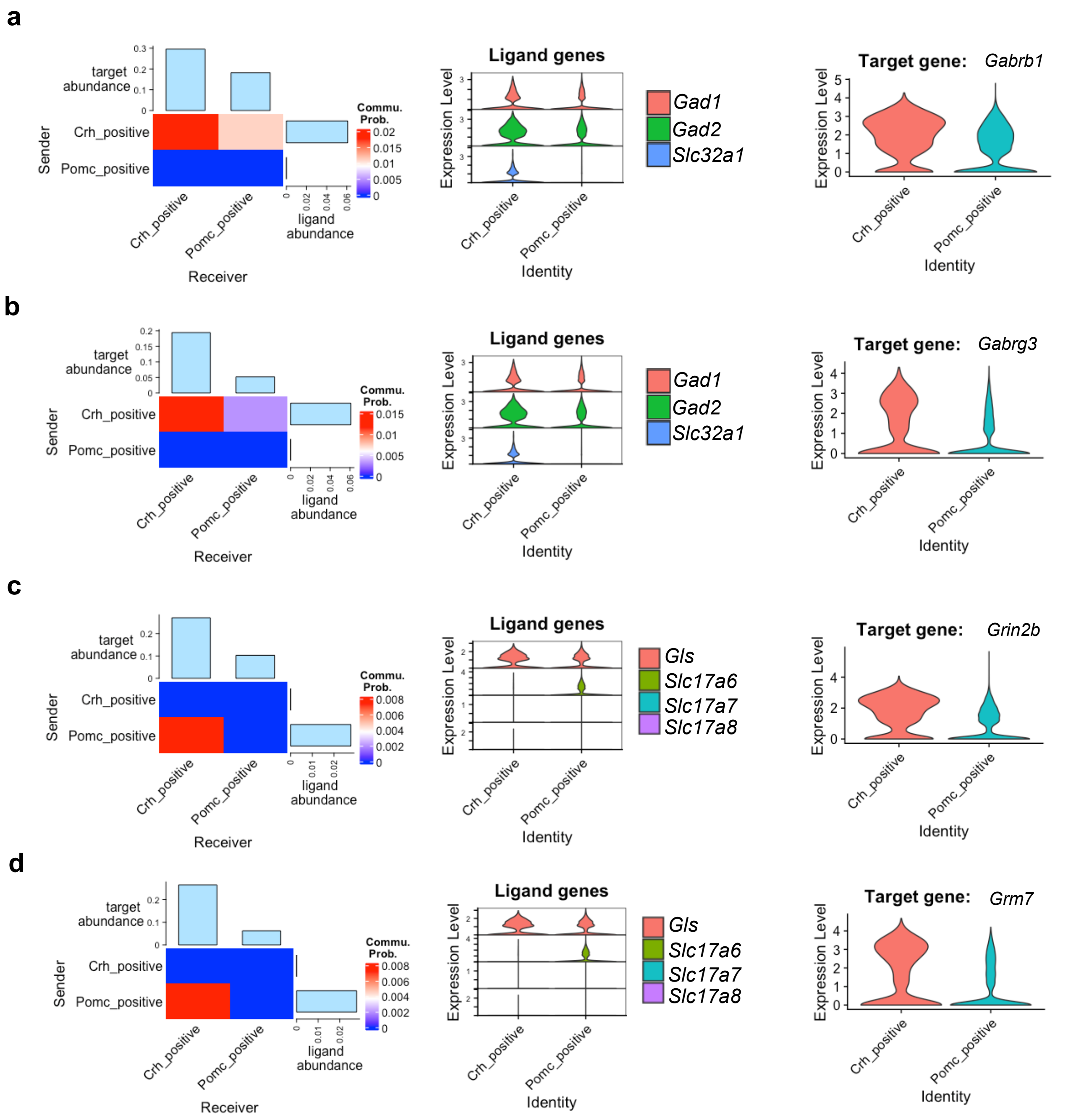

Figure S3. Comparative GABAergic and glutaminergic signaling analyses between POMC neurons-CRHNs from 17 integrated datasets. (a-b) The individual plots for the GABAergic ligand-receptor pair show that GABAergic signaling has low abundance in POMC-positive neurons compared to CRH-positive neurons, suggesting CRHNs may accept inhibitory inputs from other types of neurons (c-d), while glutaminergic signaling shows high ligand abundance from POMC neurons to CRHNs.

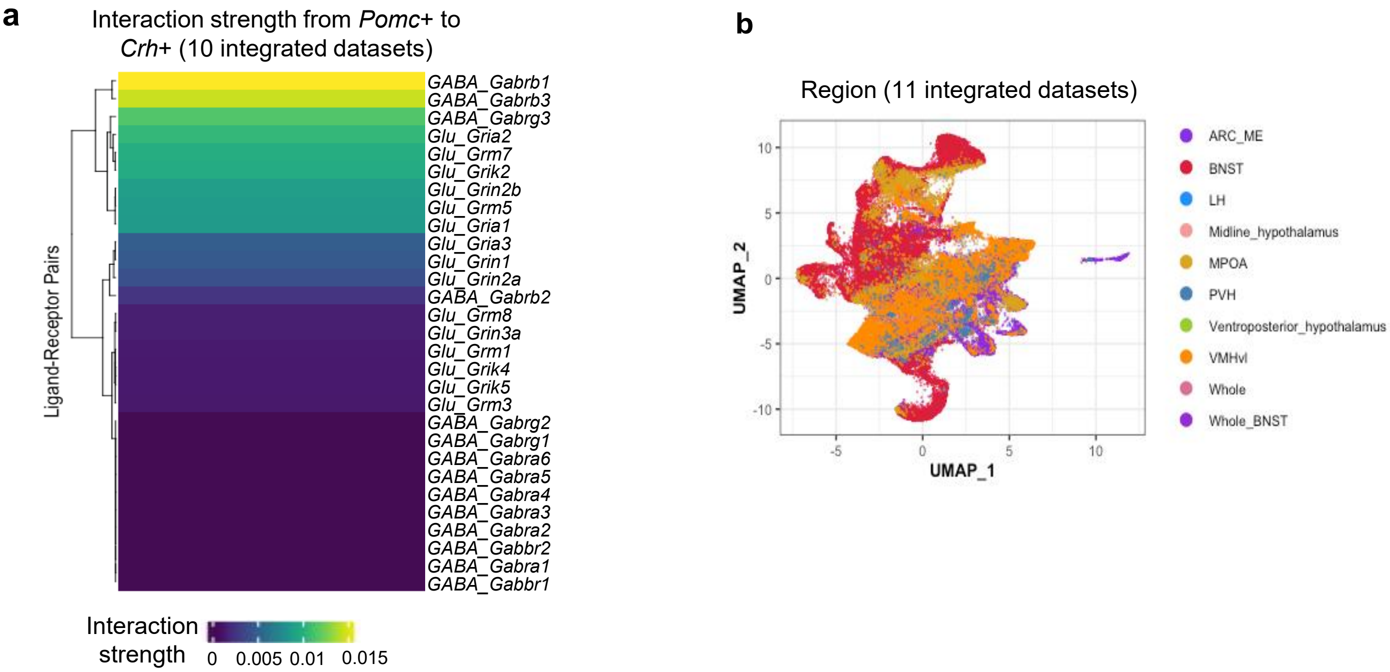

Figure S4. (a) Heatmap displays the interaction strength of ligand-receptors from POMC to CRHNs across 10 datasets (excluding PVN datasets), the color intensity indicates the strength of the interaction by glutaminergic receptors and GABAergic interactions. (b) UMAP plot with the cell colored by their receptive anatomical region from the 11 integrated datasets (adding one PVN dataset to the previous 10 datasets).
